# Clinical Scales and Wearable Sensors in Patients with Essential Tremor Treated with Deep Brain Stimulation

**DOI:** 10.1002/mdc3.70735

**Published:** 2026-07-08

**Authors:** Elena Jiltsova, Anna Hauffman, Dag Nyholm

**Affiliations:** ^1^ Department of Medical Sciences, Neurology, Uppsala University Uppsala University Hospital Uppsala Sweden; ^2^ Department of Surgical Sciences, Uppsala University Uppsala University Hospital Uppsala Sweden

**Keywords:** ambulatory monitoring, deep brain stimulation, essential tremor, tremor assessment, wearable sensors

## Abstract

**Background:**

Deep brain stimulation (DBS) for essential tremor (ET) is commonly evaluated using tremor scales, which capture only snapshot of symptoms and miss daily variability. Wearable sensors enable objective monitoring and may offer complementary information to support clinical decisions.

**Objectives:**

To investigate whether continuous wearable monitoring provides additional information to traditional analysis in ET.

**Methods:**

Forty‐five patients with ET undergoing DBS were assessed preoperatively and 9–12 months postoperatively using TETRAS scale, the EQ‐5D‐3L, and the Personal KinetiGraph (PKG) which quantified percentage time with tremor (PTT). Pre‐ and postoperative measures were compared and correlated.

**Results:**

DBS significantly improved outcomes: median TETRAS scores decreased by 55% and PKG PTT by 59%. Individual responses varied, and TETRAS and PTT showed week correlation, indicating that instruments capture different aspects of the disorder.

**Conclusions:**

Combining clinical scales with wearable monitoring complements traditional assessments by capturing daily tremor fluctuations and supporting optimized DBS management.

Essential tremor (ET) is an isolated tremor syndrome with bilateral action tremor mainly affecting upper limbs, though other regions may also be involved.^1^ Prevalence increases with age and approaches 5% of individuals older than 65 yeas.^2^ Although not life‐threatening, ET is progressive disorder and frequently associated with substantial functional impairment and reduced health‐related quality of life (HRQoL).^3^ Patients report difficulties with eating, drinking, handwriting, use of digital devices, as well as social embarrassment and occupational limitations.^4^

Pharmacological treatment is the first‐line therapy, but efficacy is limited and associated with side effects.^5^, ^6^ Deep brain stimulation (DBS) is an established treatment of medication refractory cases.^7^, ^8^ While DBS effectively reduces tremor in most patients, programming remains challenging and time‐consuming, particularly after introduction of segmented leads.^9^ Tremor severity may also fluctuate across environments and over time.^10^, ^11^

Tremor assessment relies primarily on clinical observations together with rating scales during outpatient visits.^12^, ^13^, ^14^ These measures provide important standardized evaluations but may not fully capture tremor burden. Programming decisions depend on patients’ subjective recollections of symptom control, which introduces variability and uncertainty.^15^ A reliable evaluation of the response to an intervention, eg, DBS treatment, would be advantageous.

Wearable sensor technologies allow tremor monitoring in patients’ familiar environment. The Personal KinetiGraph (PKG)^16^ has been widely implemented in Parkinson's disease.^17^, ^18^ The PKG offers continuous objective measurements of tremor and may have the potential to provide additional information to optimize DBS treatment also in ET.

The aim of this study was to evaluate usability and potential of PKG as an addition to traditional movement analysis.

## Methods

### Study Design

This prospective observational study included patients with ET referred to our center for DBS. Between 2017 and 2021, 161 patients underwent DBS implantation with a segmented lead system. Of these, 89 patients had a primary diagnosis of ET and were invited to participate in the study. Seventy patients provided written informed consent, and complete paired datasets were available for 45 patients, who constituted the final study cohort.

The diagnosis of ET was confirmed clinically by a movement disorder specialist. Novel DBS leads were implanted according to routine clinical practice. Most patients underwent unilateral surgery, while 18 received bilateral stimulation. DBS stimulation was initiated approximately 4–5 weeks after surgery. Patients were followed by the DBS team during the first postoperative year, and stimulation parameters were adjusted to optimize tremor control and minimize side effects.

### Clinical and Patient‐Reported Assessments

Baseline assessments were performed prior to DBS surgery, and follow‐up evaluations were conducted 9–12 months after implantation during stable stimulation settings. Tremor severity was evaluated using the Essential Tremor Rating Assessment Scale (TETRAS),^14^ which includes a patient‐reported activities of daily living (ADL) subscale and an observer‐rated performance subscale. All clinical assessments were performed by a DBS‐specialized nurse.

HRQoL was assessed using the EQ‐5D three‐level questionnaire (EQ‐5D‐3L),^19^ which evaluates five dimensions of health (mobility, self‐care, usual activities, pain/discomfort, and anxiety/depression) and includes a visual analogue scale (VAS) for self‐rated overall health.

### Wearable Monitoring Sensor

Objective tremor monitoring was performed using the Personal KinetiGraph (PKG; Empatica).^16^ Tremor was summarized as Percentage of Time with Tremor (PTT), representing the proportion of time when tremor was detected during monitoring over a six‐day period. Recordings were obtained from the most affected arm both before surgery and at follow‐up.

### Statistical Analysis

Only complete paired datasets were included. Data are presented as medians with interquartile ranges due to non‐normal distribution. Changes after DBS were analyzed using the Wilcoxon matched‐pairs signed‐rank test. Due to non‐normal distribution, non‐parametric analyses were used. Associations between clinical and wearable measures were assessed using Spearman's rank correlation. Statistical analyses were performed in GraphPad Prism (version 11), with significance set at *P* < 0.05 (two‐tailed).

### Ethical Considerations

The study was approved by the Swedish Ethical Review Authority Dnr. 2016/284, and all participants provided written informed consent in accordance with the Declaration of Helsinki.^20^

## Results

The cohort consisted of 45 patients with median age of 68 years at the time of surgery (Table 1). Almost all had previously tried pharmacological treatment for tremor, most commonly propranolol (41/45, 91%). By the time of referral 15 patients continued treatment. Overall, 38 patients had tried two medications or more, with side effects and limited benefits being the most common reason for discontinuation. At surgery, 20 patients remained on tremor medication, decreasing to 14 at follow‐up.

**TABLE 1:** Demographic data

Variable	Value
Participants, *n*	45
Age at surgery, years (min‐max)	68 (25–84)
Male, *n* (%)	32 (71)
Female, *n* (%)	13 (29)
Surgery bilateral, *n* (%)	18 (40)
Surgery unilateral, *n* (%)	27 (60)
Ongoing pharmacological treatment for tremor at time of surgery, *n* (%)	20 (44)
History of pharmacological treatment for tremor, *n* (%)	44 (98)
Pharmacological treatment for tremor at follow‐up, *n* (%)	14 (31)

*Note*: Follow‐up occurred at 9–12 months after surgery.

### Tremor Assessment (TETRAS and PKG)

**Figure 1:**
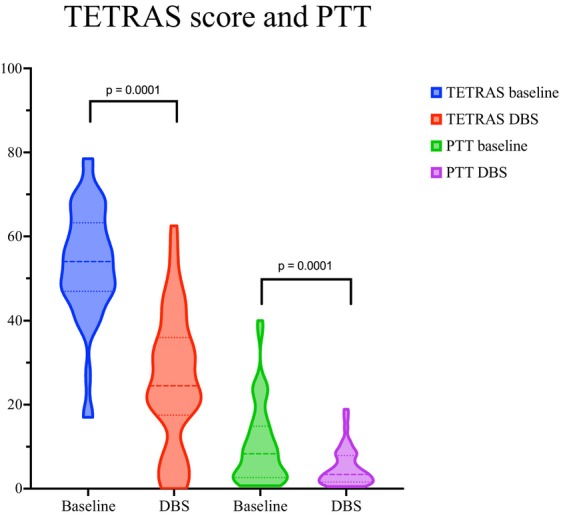
Parameters for the Essential Tremor Rating Assessments Scale (TETRAS) and Percent Time with Tremor (PTT) before and after treatment with deep brain stimulation, displayed as median with IQR, *n* = 45.

DBS markedly reduced tremor severity. Median TETRAS total scores decreased from 54% to 24.5, 55% improvement (*P* = 0.0001; Fig. 1). ADL subscores improved from 29 to 10 (66%), and performance subscores from 24.3 to 13.5 (44%), see Table S1.

PKG data showed a reduction in PTT from 8.4% to 3.4, 59% (*P* = 0.0001; Fig. 1).

At baseline, TETRAS and PTT showed a moderate correlation (Spearman's rho = 0.35), slightly stronger at follow‐up (rho = 0.47). However, the correlation between individual percent changes in TETRAS and PTT was week (rho = 0.12).

### Health Status Assessment (EQ‐5D‐3L)

The EQ5D results before and after DBS are summarized in Table S2. The most pronounced improvement in patient‐reported functioning was observed in the dimension usual activities, where a higher proportion reported no problems after DBS (from 39 to 80% on Level 1).

At baseline, 41 patients completed the VAS (median 60.0, IQR 50.0–71.5), and 40 at follow‐up (median 75.0, IQR 51.5–83.8). The mean health score increased by 6.49 points, with 62.5% showing higher VAS scores at follow‐up, 30.0% lower scores, and 7.5% no change (Fig. S1).

## Discussion

In this prospective observational study, we combined a traditional clinical tremor rating scale (TETRAS) with continuous ambulatory monitoring using a wearable sensor (PKG) in patients with ET, before and after DBS treatment. To our knowledge, the integration of continuous ambulatory monitoring via the PKG sensor to complement rating scale findings represents a novel approach in this patient population. Our results demonstrated a substantial improvement in tremor severity following DBS, assessed by both clinical scales and by objective wearable monitoring. The magnitude of improvement recorded with TETRAS is consistent with previously reported outcomes for DBS in ET^22^. Importantly, PKG recordings also demonstrated a reduction in tremor occurrence at the group level, supporting clinical observations of treatment efficacy in everyday life. Despite similar group‐level improvements, individual patients often demonstrated different responses in TETRAS and PTT measurements. The weak correlation between clinical tremor scores and PTT suggests that these methods capture different dimensions of tremor burden. TETRAS provides a structured snapshot of tremor severity, whereas the PKG quantifies the temporal occurrence of tremor during daily life. Improvements observed during clinical assessments may therefore not fully translate into proportional reductions in tremor occurrence during everyday activities. Moreover, patients with improvement after DBS may resume previously avoided activities, which can lead to even increased PTT values despite better function. This illustrates the potential of wearable monitoring to provide important complementary information about tremor response outside clinical settings.

Patient‐reported outcomes demonstrated modest improvements in the EQ‐5D domain usual activities, whereas mobility remained largely unchanged. This finding is consistent with the expected effects of DBS in essential tremor, which primarily targets action tremor rather than gait or balance. Despite substantial tremor reduction, some patients reported limited improvement in overall quality of life on VAS scale, illustrating that quality‐of‐life outcomes are influenced by multiple factors beyond tremor control. Moreover, clinically meaningful difference for EQ‐5D have not been clearly established in this population, limiting the interpretability of the observed changes.

Optimization of DBS stimulation parameters represents an important challenge in the long‐term management of patients. Programming is time‐consuming and often requires repeated clinical evaluations, particularly with the introduction of segmented leads that substantially expand number of possible stimulation settings. Current programming strategies rely on in‐clinic assessments and patient recall, which may not fully capture symptoms fluctuations during daily life. Objective ambulatory monitoring therefore has the potential to provide additional information during the DBS optimization process. Although the PKG system was originally developed and validated for monitoring motor symptoms in Parkinson's disease, its ability to detect tremor allows pragmatic application in patients with ET. The smartwatch was well tolerated by patients and provided a robust estimate of tremor presence over time. Together with clinical scales, the two methods may provide a more comprehensive understanding of treatment response.

This study has several limitations. First, this was an observational study without a control group, limiting causal inference. Second, although the cohort was relatively large for a DBS study in ET, complete datasets were available for only 45 of 70 participants, partly due to data collection during the COVID‐19 pandemic. Third, the Personal KinetiGraph was originally developed and validated in patients with Parkinson's disease and has not been specifically validated for ET. Differences in tremor characteristics between Parkinson's disease and ET may therefore influence the interpretation of PKG‐derived tremor metrics in this population. The present study was not designed to validate PKG as an independent measure of tremor severity in ET, but rather to explore the potential complementary role of continuous ambulatory monitoring alongside established clinical assessments. Finally, outcomes were assessed at a single postoperative time point, and longer follow‐up with repeated assessments would be required to evaluate temporal changes in treatment response, including potential habituation or loss of DBS efficacy.

A key strength of this study is the integration of established clinical tremor rating scales with a wearable sensor technique to evaluate the effect of DBS. Combining the two approaches provides a more comprehensive assessment of tremor burden, treatment response, and may eventually facilitate optimization of DBS stimulation parameters.

In conclusion, while wearable sensors are not designed to replace bedside evaluations, they offer valuable insights into the real‐world burden of tremor and the effects of treatment. Complementary monitoring outside the hospital setting is well tolerated by patients and may facilitate more informed clinical decision‐making regarding further optimization of DBS therapy for ET. The continued development of wearable devices, with the capability for detailed analysis of tremor, can assist clinicians in optimizing reprogramming efforts more objectively and potentially establish protocols for home monitoring of treatment modifications in an environment familiar to the patient. This approach could better elucidate the potential of DBS as a technique and provide greater benefits for patients.

## Author Roles

(1) Research project: A. Conception, B. Organization, C. Execution; (2) Statistical Analysis: A. Design, B. Execution, C. Review and Critique; (3) Manuscript Preparation: A. Writing of the first draft, B. Review and Critique

E.J.: 1A, 1B, 1C, 2A, 2B, 2C, 3A, 3B

A.H.: 2B, 2C, 3A, 3B

D.N.: 1A, 1B, 2C, 3B

## Disclosures

**Ethical Compliance Statement:** The authors confirm this work has been approved by Swedish Ethical Review Authority and every patient gave written informed consent to participate in the project. The authors confirm that we have read the Journal's position on issues involved in ethical publication and affirm that this work is consistent with those guidelines.

**Financial Disclosures for the Previous 12 Months:** The authors declare that there are no additional disclosures to report.

## Financial Disclosures and Conflicts of Interest

Author disclosures are available in the Supporting Information.

## Supporting information


**Figure S1.** EQ‐5 D‐3 L VAS distribution before and following Deep Brain Stimulation *n* = 40 Please note: some individual baseline‐ and follow‐up values overlap in the figure.
**TABLE S1.** Tremor severity before and after DBS (*n* = 45).
**TABLE S2.** Frequencies for the EQ‐5D three‐level version for those with complete datasets at baseline and after treatment with Deep Brain Stimulation.

**Data S1.** COI_disclosure

## Data Availability

The data that support the findings of this study are available on request from the corresponding author. The data are not publicly available due to privacy or ethical restrictions.
