# Supplementary material for: Clinical Scales and Wearable Sensors in Patients with Essential Tremor Treated with Deep Brain Stimulation
Source: Mov Disord Clin Pract. 2026 Jul 8:10.1002/mdc3.70735. Online ahead of print. doi: 10.1002/mdc3.70735 (PMC13346013; doi:10.1002/mdc3.70735)
Supplement: Supplementary file 1 — Figure S1. EQ‐5 D‐3 L VAS distribution before and following Deep Brain Stimulation n = 40 Please note: some individual baseline‐ and follow‐up values overlap in the figure. TABLE S1. Tremor severity before and after DBS (n = 45). TABLE S2. Frequencies for the EQ‐5D three‐level version for those with complete datasets at baseline and after treatment with Deep Brain Stimulation. [file MDC3-9999-0-s001.docx]

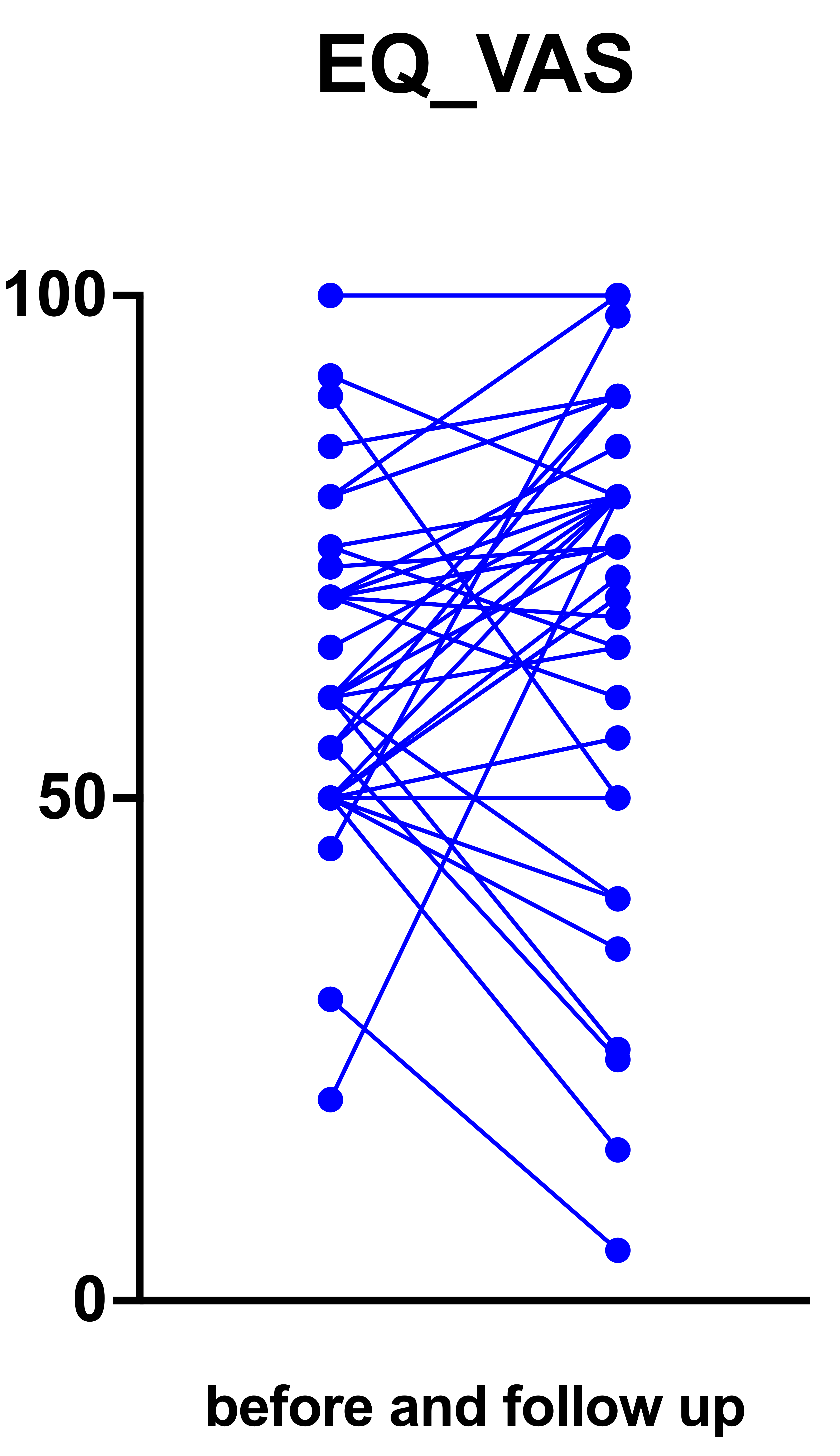


Supplementary Figure 1. EQ-5 D-3L VAS distribution before and following Deep Brain Stimulation n= 40 Please note: some individual baseline- and follow-up values overlap in the figure

Supplementary Table 1. Tremor severity before and after DBS (n=45)

| **Measure** | **Baseline, median (IQR)** | **Follow-up, median (IQR)** |
| --- | --- | --- |
| TETRAS Total median (IQR) | 54 (47-63.3) | 24.5 (17.5-36.0) |
| TETRAS ADL median (IQR) | 29.0 (24.9-33.4) | 10.0 (5.0-21.8) |
| TETRAS Performance median (IQR) | 24.1 (20.0-31.1) | 13.5 (8.9-19.5) |
| PKG (PTT*) median (IQR) | 8.3 (2.7-14.9) | 3.4 (1.6-7.9) |

Please note: *PTT= Percentage of Time with Tremor

Supplementary Table 2. Frequencies for the EQ-5D three-level version for those with complete datasets at baseline and after treatment with Deep Brain Stimulation

| **No (%)** | **Mobility** | | **Self-care** | | **Usual activities** | | **Pain/**  **discomfort** | | **Anxiety/ Depression** | |
| --- | --- | --- | --- | --- | --- | --- | --- | --- | --- | --- |
|  | **BL** | **DBS** | **BL** | **DBS** | **BL** | **DBS** | **BL** | **DBS** | **BL** | **DBS** |
| **Level 1** | 31  (76) | 28  (72) | 32  (78) | 37  (95) | 16  (39) | 31  (80) | 10  (24) | 16  (42) | 19  (47) | 27  (71) |
| **Level 2** | 10  (24) | 11  (28) | 9  (22) | 2  (5) | 25  (61) | 6  (15) | 25  (61) | 17  (45) | 21  (51) | 10  (26) |
| **Level 3** | 0 | 0 | 0 | 0 | 0 | 2  (5) | 6  (15) | 5  (13) | 1  (2) | 1  (3) |
| **Total** | 41 | 39 | 41 | 39 | 41 | 39 | 41 | 38 | 41 | 38 |

Please note: Only complete data are presented, and percentages are calculated based on the total number of respondents for each dimension (n= 38-41). Level 1: indicating no problems, Level 2: indicating some problems, Level 3: indicating extreme problems. Abbreviations: BL=Baseline, DBS=Deep Brain Stimulation
